# Seed Size, Fecundity and Postfire Regeneration Strategy Are Interdependent in *Hakea*

**DOI:** 10.1371/journal.pone.0129027

**Published:** 2015-06-02

**Authors:** Sh-hoob Mohamed El-ahmir, Sim Lin Lim, Byron B. Lamont, Tianhua He

**Affiliations:** Department of Environment and Agriculture, Curtin University, Perth, WA, Australia; University of Florida, UNITED STATES

## Abstract

Seed size is a key functional trait that affects plant fitness at the seedling stage and may vary greatly with species fruit size, growth form and fecundity. Using structural equation modelling (SEM) and correlated trait evolution analysis, we investigated the interaction network between seed size and fecundity, postfire regeneration strategy, fruit size, plant height and serotiny (on-plant seed storage) among 82 species of the woody shrub genus, *Hakea*, with a wide spectrum of seed sizes (2–500 mg). Seed size is negatively correlated with fecundity, while fire-killed species (nonsprouters) produce more seeds than resprouters though they are of similar size. Seed size is unrelated to plant height and level of serotiny while it scales allometrically with fruit size. A strong phylogenetic signal in seed size revealed phylogenetic constraints on seed size variation in *Hakea*. Our analyses suggest a causal relationship between seed size, fecundity and postfire regeneration strategy in *Hakea*. These results demonstrate that fruit size, fecundity and evolutionary history have had most control over seed size variation among *Hakea* species.

## Introduction

Seed size is a key trait in the life history of plants that affects fitness at the seedling stage and is often correlated with other attributes important in their evolution and ecology [1]. Seed size among angiosperms varies from 1 × 10^–5^ g to 3 × 10^4^ g [2]. Many factors have been shown to influence seed size, such as resource availability [3, 4], growing conditions [5,6], and plant growth form, longevity and height [7, 8]. For example, by analysing seed mass data for 13,000 species, Moles et al. [8] concluded that there is a close association between seed size and plant height that is likely the result of the scaling of seed size to plant height [9]. In addition, seed size variation may also be subject to phylogenetic constraints on seed development, such that closely related species may have similar seed sizes [9].

Recurrent fire is a prominent phenomenon in ecosystems with Mediterranean-type climates, such as those in southwestern Australia (SWA). Recent research points to a significant role for fire in shaping the evolution of plant functional traits in these fire-prone ecosystems [10, 11]. However, studies of how fire might have influenced seed size variation are scarce. Plants in fire-prone ecosystems can be divided into different functional groups in terms of their overall response to fire. In the simplest scheme, the fire response of plant species entails nonsprouters (killed by fire, and populations regenerate solely from seedlings) and resprouters (resprout after fire from roots, rhizomes, lignotubers or major stems of the pre-fire plants) [12, 13, 14]. This divergence of life form and postfire regeneration strategy in fire-prone environments can be expected to have significant implications for seed size variation through direct or indirect interactions.

Carpenter and Recher [15] first proposed that fire-response strategies are linked with reproductive features, such as fecundity. Nonsprouters should invest more resources in seed production than do resprouters because resprouters have the ability to survive via self-replacement. By comparing species pairs, Lamont and Wiens [13] showed that nonsprouting species indeed have greater seed set on a per ovule basis than resprouters, but it is by no means universal [16, 17]. An improvement in resource availability usually leads to greater seed production mainly because the plants are larger [18, 19], but the reverse may also be true [20]. In addition, there is much support for a trade-off between fecundity and seed size [21, 22, 23], though this relationship must be set in the context of other life-history traits. Working in fire-prone sclerophyll shrublands, Esther et al. [24] showed that the two most important interactions affecting population viability were seed size–seed production and seed size–regeneration strategy. Resprouters always did well, but the success of nonsprouters depended on their having many or large seeds.

Nonsprouters might opt for many small seeds as these have a greater probability of reaching favourable habitats further from the parents than larger seeds [25]. Since small seeds produce small seedlings they are likely to be more drought-prone [26, 27]. Where both fire-response types produce few seeds they are expected to be larger as seedling survival is dependent on quickly developing a strong root system, possible only from larger seeds, to avoid the effects of drought [28, 29]. Heavy seeds may also gain a competitive advantage over small seeds due to their earlier germination [30]. Resprouters typically produce few seeds, and they invest less in reproductive organs relative to the storage functions that help them re-establish quickly after fire, irrespective of seed size, and thus there should be a discernable relationship between fire response and seed size.

Serotiny (prolonged storage of seeds on the plant) is characteristic of fire-prone, sclerophyll vegetation worldwide [31]. Mature seeds are retained in the crown and seed release is usually cued by heat from fire. Empirical observations suggest serotinous species usually produce large fruits [31, 32]. Serotinous seeds take longer to mature (1–3 years) and therefore can receive more resources during seed filling [31, 32, 33]. Secondly, serotinous species usually have large, woody fruits on stout stems to protect their seeds against predators and temperature extremes. Given a fixed number of seeds per fruit (e.g., two seeds in each follicle), larger fruits can support and nurture larger seeds.

The endemic Australian genus *Hakea* (Proteaceae) is known for its wide range of seed sizes (2–500 mg) among its 150 extant species [34], 100 of which inhabit the nutrient-impoverished soils of southwestern Australia (SWA), characterised by hot, dry summers and frequent fire [35]. Species are either killed by fire or resprout from lignotubers or sometimes epicormic buds or lateral roots [36]. Growth form varies from creeping sub-shrubs to trees rarely >5 m tall. All possess woody fruits that vary in size by >3 orders of magnitude and in degree of serotiny from zero to ~10 years [37] and on-plant seed storage varies from close to zero (some resprouters) to thousands (large nonsprouters) of seeds [34, 36]. Much study have looked into the relationship of fruit size and seed size [37], fruit size and postfire regeneration strategy [36], serotiny and fruit size [33], and generated significant insights into the ecology of seed size variation in *Hakea*. However, as the majority of those studies investigated a simple relationship between seed size and another functional trait, it is not clear how these functional traits interact in a network of ecological setting and in an evolutionary context.

In this study, we used structural equation modelling (SEM) analysis and correlated trait evolution analysis to explore the interacting network of seed size, postfire regeneration strategy, fecundity, fruit size, serotiny and plant height in a phylogenetic context including 82 species. Our objective was to identify the driving force behind variation in seed size within a genus adapted to poor soils, recurrent fire and severe summer drought.

## Material and Methods

### Trait data and structural equation modelling analysis

We focused on seed size and five functional and life history traits that are expected to influence seed size in *Hakea*. Trait data were collated from the literature [34, 35, 38, 39, 40, 41]. A total of 82 species covering the genus morphological variation and distribution range, and with relatively even numbers of resprouters and nonsprouters, were investigated (S1 Table).

**Fig 1:**
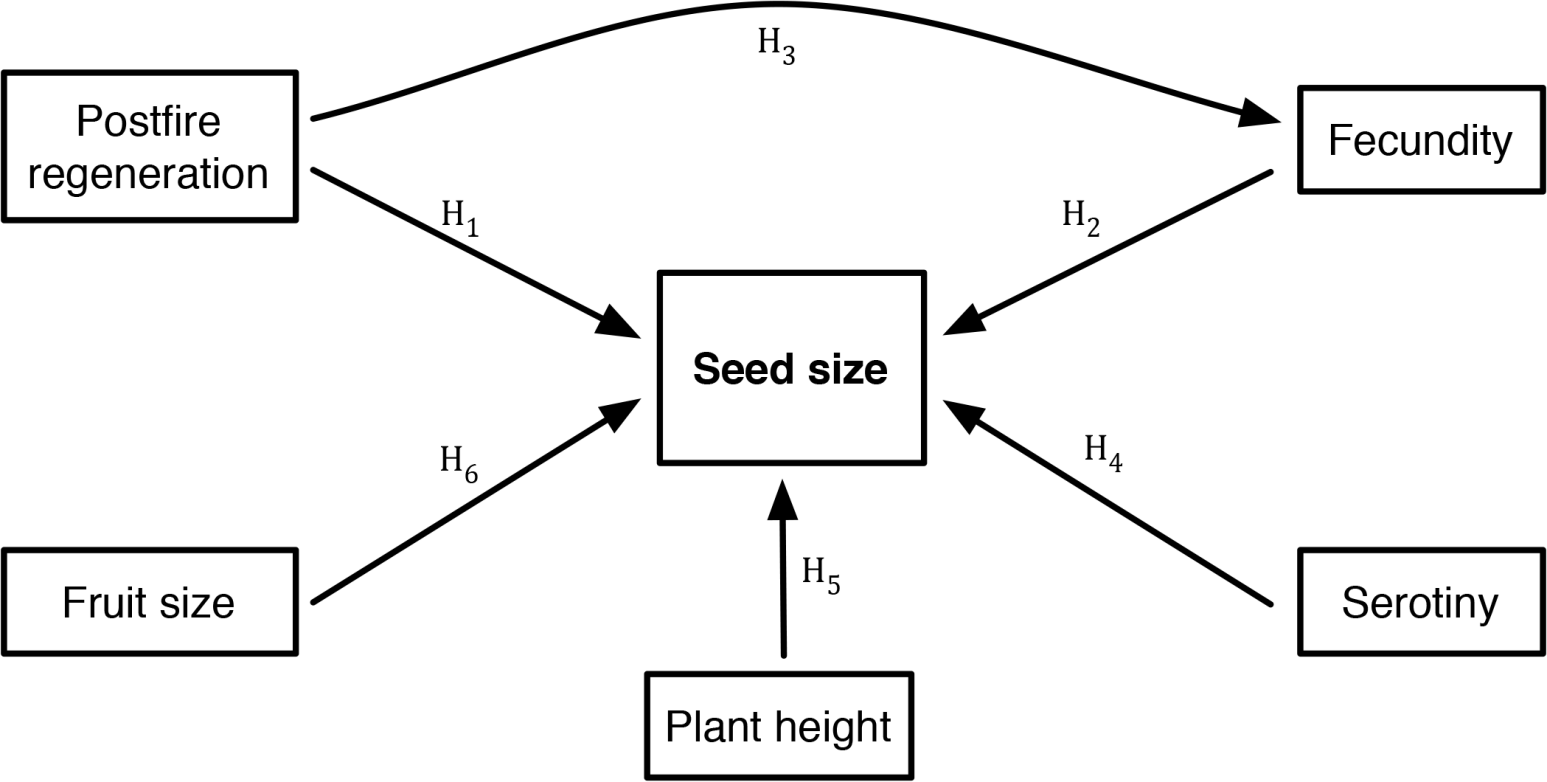
Conceptual model showing working hypotheses on the interaction between seed size and other functional and life history traits. See text for hypotheses H_1_–H_6_.

We first used Structural Equation Modelling (SEM) analysis to generate and explore models that infer the causal relationships between seed size and putative interacting traits. SEM extends the basic correlation approach to path analysis by directly testing the goodness of fit of the model to the data, calculates correlation coefficients, and separates total effects into direct and indirect effects [42]. Models can be modified by deleting pathways that are not correlated, therefore optimising the fit of the model. The modelling process in SEM analysis is based on *a priori* and theoretical knowledge and begins with a consideration of expected relationships based on the mechanisms predicted to operate in the system. We began by building a conceptual SEM model of the expected multivariate relationships based on prevailing theory of the interactions between seed size and functional or life history traits, and then refined the model by deleting the uncorrelated pathways. Seed size and another five functional or life history traits for each of the 82 species were included in the SEM model (Fig 1): 1) plant height, 2) postfire regeneration strategy, 3) fecundity (on-plant seed store), 4) serotiny, and 5) fruit size. Fecundity was estimated as the number of fruits stored on plants at least 15 years since the last fire. Each fruit supported two seeds though very occasionally one of these may abort. Seed and fruit size (dry mass) were continuous data while height, regeneration strategy, fecundity and serotiny were categorical. The working hypotheses were based on the following predictions:

H_1_: Nonsprouters produce more seeds or larger seeds than resprouters [24], and have greater investment in seeds [13, 25, 30, 29];
H_2_: There is a negative correlation between fecundity and seed size [21, 22, 23];
H_3_: Resprouters have lower fecundity [15], and therefore a larger trade-off in resource limited systems;
H_4_: Strongly serotinous species produce larger seeds than non-weakly serotinous species [31, 32, 33];
H_5_: Taller plants produce larger seeds [8, 9];
H_6_: Larger fruits possess larger seeds (since all fruits contain two seeds) as suggested by allometric logic [43].

SEM was performed in SPSS AMOS 18.0.0 (Analysis of Moment Structures, SPSS Inc., Chicago, USA). The conceptual model was examined using a likelihood approach, and non-significant pathways were later deleted, and the model with the remaining pathways was retested. The significance of correlations was taken as one-tailed, *P* ≤ 0.05 because our predictions were directional.

### Phylogenetic analysis of correlated evolution between seed size and other traits

Trait correlations were further tested in a phylogenetic context with divergence time as branch length. We built a *Hakea* phylogeny of the 82 species using gene sequences extracted from NCBI (51 species), combined with new sequences generated in this study (31 species). The newly generated sequence for each species we amplified 8 DNAregions: the nuclear ribosomal internal transcribed spacers (ITS) and plastid *matK*, *rbcL*, *trnL* intron, and *trnL-trnF* intergenic spacer, *atpB*, *atpB-rbcL* intergenic spacer, and *rpl16* intron, were produced following standard protocols (GenBank accession numbers shown in S2 Table) [44]. *Grevillea juncifolia*, *Finchia chloroxantha*, *Buckinghamia celsissima*, *Banksia serrata* and *Persoonia lanceolata* (all Proteaceae) DNA sequences were chosen as outgroup for the *Hakea* phylogenetic analysis (S2 Table). The sequences were aligned and edited using the computer software MUSCLE [45].

BEAST v2.1.0 [46] was used to estimate phylogeny relationships and divergence time under a strict clock model [47] that provided phylogenetic topology consistent with previous studies [41, 44]. The dataset was partitioned by genes, with each partition unlinked and set to a general time reversible (GTR) model with γ-distributed rate heterogeneity. We set the calibration point for crown Proteaceae at 70.6 My as suggested by Sauquet [44] based on the fossil *Lewalanipollis rectomarginis* described by Khan [48]. We used a Yule prior for rates of cladogenesis and ran analyses of 10 million generations, sampling every 1000 generations. The program Tracer [49] was used to visualize the posterior distribution of trees and estimate the appropriate burn-in. Consequently, a 2.5 million generation burn-in was determined. The details and settings of generating BEAST phylogeny is provided as an xml file in the supplementary material. TreeAnnotator v1.6.1 [46] was used to generate a maximum credibility tree (MC tree) based on this analysis.

The degree of phylogenetic signal in the six traits in *Hakea* was tested using Pagel’s lambda (λ) based on 1000 *Hakea* phylogenies generated from BEAST in above analysis. A value of 0 indicates no significant phylogenetic signal in the trait, while a value of 1 indicates complete phylogenetic patterning. Pagel’s λ estimation and significance tests were conducted in the R package ‘Geiger’ [50].

BayesTraits continuous random walk (Model A) was used to determine the relationships between pairwise *Hakea* traits, as illustrated in the conceptual model [51]. BayesTraits uses a Monte Carlo Markov Chain (MCMC) procedure to calculate the harmonic means of different pairs of *Hakea* traits based on the MC tree. Bayes factors (BF) were used to determine IF significant phylogenetic correlations between two traits (BF < 2: weak; 2 >BF < 5: moderate; BF 5~10: strong). Our study excluded the outgroup taxa in these analyses to avoid introducing bias in estimates of trait relationships that might occur when a single taxon is used to represent a much larger group [52].

## Results

Seed size showed wide variation among the 82 *Hakea* species, and both resprouters and nonsprouters had a wide range of seed weights. For example, among resprouters, *H*. *oleifolia* seeds weigh 5 mg while *H*. *flabellifolia* seeds weigh >156 mg. The nonsprouting *H*. *sulcata* has a seed weight of 3 mg but *H*. *platysperma* weighs >509 mg. However, resprouting species had lower fecundity than nonsprouters when adjusted for plant size. Nonsprouting species produced on average more than 100 fruits per plant, while resprouters averaged half this number.

**Fig 2:**
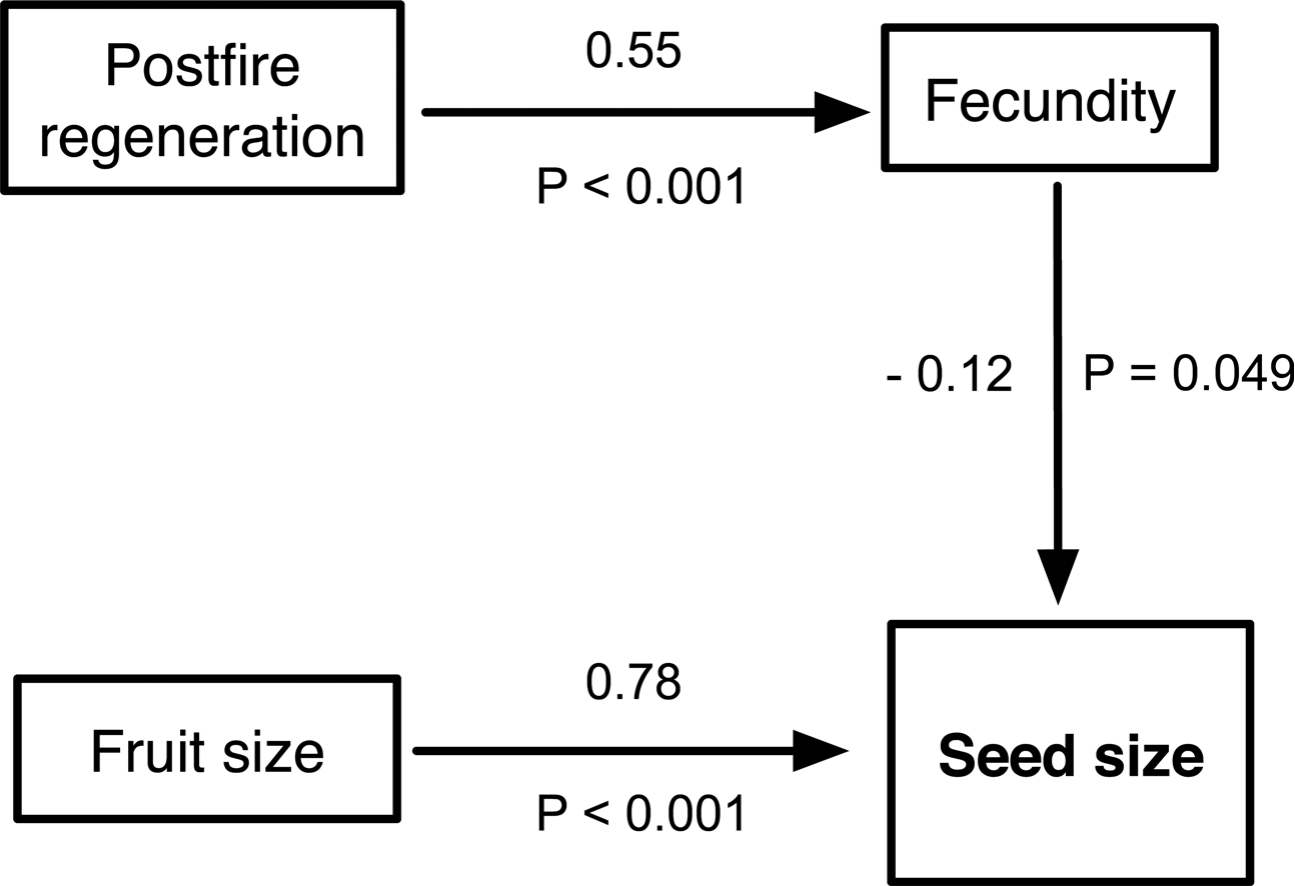
Simplified structural equation modelling analysis showing the significant interacting pathways between seed size, fecundity, postfire regeneration strategy and fruit size. Numbers above the lines are the standardised direct effects.

Seeds of resprouting species were slightly lighter than that of nonsprouters (34.5 ± 34.1 mg vs 40.0 ± 76.6 mg, mean ± standard deviation, respectively), but fire response had no direct effect on seed size variation in *Hakea* (*P* = 0.471; Table 1). Larger seeds were not associated with taller plants (*P* = 0.262), and serotinous species did not necessarily have larger seeds than weakly- or non-serotinous species (*P* = 0.240; Table 1). Deleting these non-significant pathways, the final SEM analysis revealed a direct causal correlation between the postfire regeneration strategy and fecundity (resprouters store fewer seeds) with a direct effect of 0.55 (*P* < 0.001), and a significant trade-off between fecundity and seed size (direct effect = -0.12; *P* = 0.047), such that species with more seeds had smaller seeds. Strong positive correlations were observed between fruit size and seed size with a direct effect of 0.78 (*P* < 0.001), i.e., heavier fruits have larger seeds (Fig 2).

**Table 1:** Standard direct effect and associated probability of the hypothesised interaction pathways in the conceptual model. (*) Star indicates hypothesis supported.

Dependent variable		H_1_	H_2_^*^	H_3_^*^	H_4_	H_5_	H_6_^*^
Seed size		Postfire response		Fecundity	Serotiny	Plant height	Fruit size
Fecundity			Postfire response				
	Standardised direct effect	0.030	0.550	-0.150	-0.058	0.044	0.779
	*P* (one-tailed)	0.360	<0.001	0.047	0.240	0.255	<0.001

**Fig 3:**
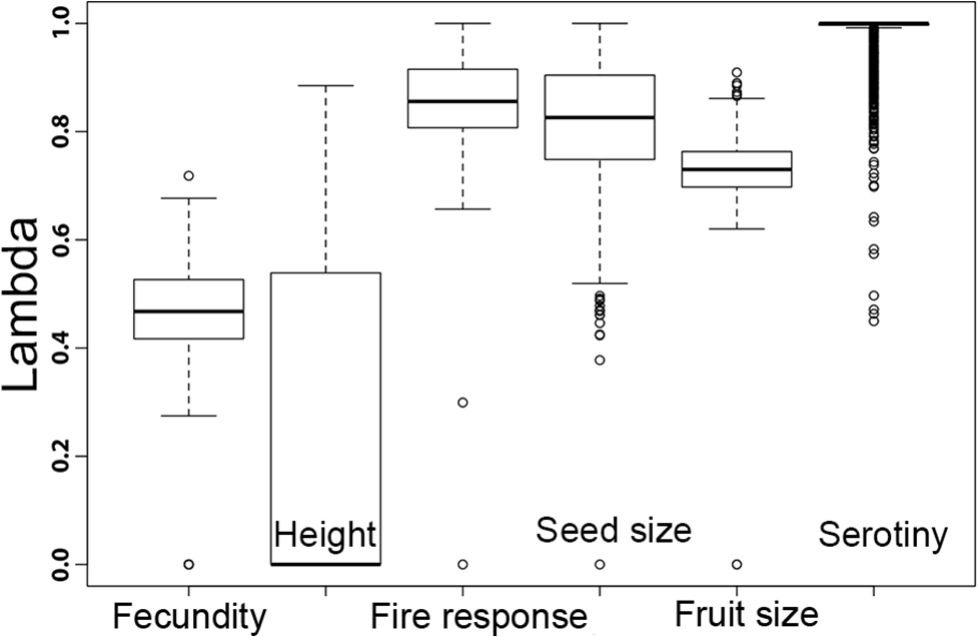
Distribution of lambda values among 1000 simulations indicating the degree of phylogenetic constraints on six traits in *Hakea*. Means are shown by thickened horizontal lines, standard deviations are bounded by boxes and ranges are connected by broken lines, and circles are outliers.

**Fig 4:**
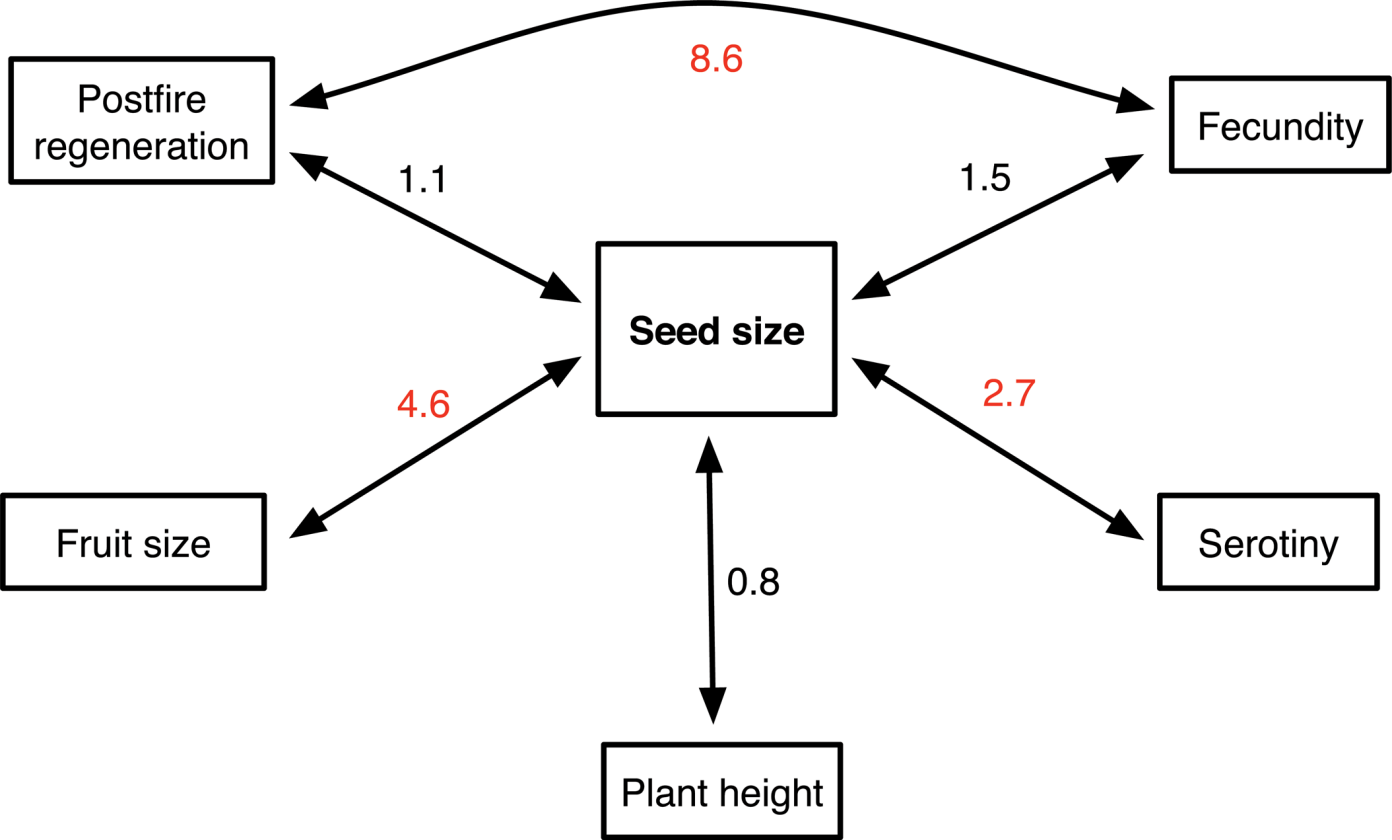
Evolutionary associations between pairwise traits in *Hakea*. Numbers beside the arrows are Bayes factors (BF), with BF > 2 indicating strong association.

The topology of our *Hakea* phylogeny, which included 82 species, was consistent with one reported earlier by Mast et al. [41] which included 55 species were included (S1 Fig). We detected a strong phylogenetic signals for seed size with a λ of 0.82, implying closely-related *Hakea* species tend to be more similar in seed size than expected by chance (Fig 3). Similar results were recovered for postfire regeneration strategy and (especially) serotiny, and, to a lesser extent, fruit size. Plant height and fecundity were less constrained by phylogeny with λ much less than one. From the trait data and time-calibrated phylogeny, associated evolution between pairwise traits was noted in *Hakea* using Bayesian MCMC analysis (Fig 4). The analysis revealed significant evolutionary correlations between postfire regeneration strategy and fecundity (BF = 8.6), and between seed size and fruit size (BF = 4.6). Seed size and serotiny are also appear to be correlated (BF = 2.7). Seed size showed a weak association with fecundity (BF = 1.5) and with postfire regeneration strategy (BF = 1.1). Plant height was unlikely to have been related to seed size during the evolution of the genus (BF = 0.8).

## Discussion

Structural equation modelling and Bayesian MCMC analysis showed that seed size was most significantly associated, in a co-evolutionary sense, with fruit size where larger fruits support larger seeds (H_6_). This relationship is clearly causal as a) the number of seeds per fruit is fixed (two) so that only seed size can vary, b) the pericarp acts directly as a source of nutrients for seed filling [33], c) larger fruits have a better vascular supply for seed filling [53], and d) larger (woody) fruits are an adaptive response to the greater vulnerability of larger seeds to granivores [32].

However, the relationship between seed and fruit size breaks down when serotiny (prolonged on-plant seed storage) is considered. Although seed size increases with stronger serotiny through evolutionary time (second only to fruit size), the follicle:seed weight ratio of strongly serotinous species is six times that of weakly serotinous species without any difference in seed weight [37]. This is not so when a wider range of genera is collated [33] therefore it must be a special feature of *Hakea*. It appears that protecting the seeds from granivorous cockatoos, during their prolonged storage on the plant, has taken precedence over any potential benefits of larger seed size. Nevertheless, Groom and Lamont [33] show that in SWA the phosphorus concentration of strongly serotinous species is 40% higher than in weakly serotinous *Hakea* species. This confirms that the seed size-nutrient content relationship is not crucial to the ability of hakeas to recruit inter-fire as occurs with weakly serotinous species, in contrast to the anti-herbivore role of their phenolic content [54].

Despite a recorded seed weight range of 2 to 500 mg, nonsprouters (38 mg) and resprouters (36 mg) had similar mean seed mass i.e., there was no relationship between regeneration strategies and seed size, therefore hypothesis H_1_ (nonsprouters produce larger seeds than resprouters) was not supported by the analysis. Just over half the species in both fire-response types had seeds weighing >20 mg, a size considered to contain sufficient nutrient resources to ensure adequate root extension for survival of the initial summer drought in the poor soils of SWA [29]. The remaining species must rely on drought-tolerant traits [27]. Seed size conservatism within a species contrasts with huge differences in seed number associated with variations in plant age and size, and nutrient and water availability [55, 31, 56, 57]. Thus it seems that a given seed size is embedded in the adaptive biology of each species by strong selection pressures (e.g., resource availability) and shows little phenotypic plasticity.

SEM analysis supported our expectation of a relationship between postfire regeneration strategies and fecundity (*P* < 0. 001, hypothesis H_2_). Further, the two traits have coevolved, as revealed by the Bayesian MCMC analysis. Given that both more and larger seeds may be adaptive among nonsprouters, as they regenerate solely from seeds after fire and their seedlings establish in nutrient-impoverished environments [27, 24], this fire-response type opts for more rather than larger seeds. Extensive demographic studies on hakeas and related woody species in SWA have shown that postfire recruitment patterns conform to biased lotteries, with the demographic component paramount followed by biotic components, such as seedling size [58]. Given a fixed seed size, the best option to ensure population viability of fire-killed species is through a large seed store. This is achieved via faster growth rates, earlier time to maturity, more flowers/plant, more seeds/ovule, higher seed viability and finally more seedlings/parent compared with resprouters [13]. In contrast, low fecundity among resprouters may be best related to the accumulation of deleterious somatic mutations, a random, time-dependent process unrelated to seed size and to which nonsprouters are immune [13] but see [59].

Using structural equation modelling and Bayesian MCMC analysis, we show that seed size is traded off with species fecundity (H_3_). For example, *H*. *flabellifolia* seeds weigh 156 mg and it produces only one or two fruits per plant. In contrast, *H*. *pycnoneura* and *H*. *scoparia* have seed weights of only 5.9 mg but >100 fruits per plant. Apart from a trade-off with fecundity, further phylogenetic analysis revealed that seed size in *Hakea* might also be constrained by speciation patterns in the genus, i.e., closely related species tend to have similar seed sizes. For example, in *Hakea*, the Ulicina group has relatively small seeds while the Ceratophylla group has large seeds [35]. Interestingly, fire response and serotiny, both considered adaptations to fire-prone environments, are shown here to have phylogenetic signals. It is likely that seed size in *Hakea* might have tracked selection pressure from fire as well. These processes are one explanation for the apparent trade-off between the size of seed stores and seed size, and phylogenetic constraints on seed size. They provide insights as to why the relationship is not strong for either of them because of the over-riding interactions with resource limitations and other selective pressures in fire-prone environments.

Global variation in seed size is associated with divergence in plant growth form [8], with taller plants supporting larger seeds, which is assumed to reflect a trade-off between likelihood of survival to maturity (low in tall plants) and offspring size. However, plant height has no direct effect on seed size in *Hakea* (H_5_). Drawing parallels with other congeneric pairs, resprouters are the slowest, and least likely, to mature but they are rarely the tallest [13, 60] and seed size is no different from nonsprouters. It is true that larger seeds have a lower wing/mass ratio than smaller seeds among hakeas [61] and thus might benefit from a greater release height. On the other hand, long-distance dispersal is facilitated by wind vortices that lift and carry seeds from the ground in postfire habitats of SWA making seed size less relevant to their dispersal potential [62, 63]. It is also worth noting that plant height variation in *Hakea* is small (0.5–5 m) and may not be sufficient to promote divergence in seed size.

In conclusion, the synthesis of powerful SEM analyses and robust phylogenies, by which multiple trait data sets are compared, revealed causal relationships between seed size and fruit size (strong) and fecundity (weak) in *Hakea*, and between fecundity and postfire regeneration strategy (strong), but not between seed size and regeneration strategy, plant stature or serotiny. Large seeds are supported/protected by large fruits and have a weak trade-off with fecundity that is much lower among resprouters even though these do not have larger seeds. All relationships are constrained to some extent by their evolutionary history, with seed size correlated with fruit size and serotiny through evolutionary time.

## Supporting Information

S1 Appendix The xml file for details and settings in generating phylogeny in BEAST. (XML)

S1 Fig *Hakea* maximum credibility phylogeny generated by BEAST. (DOCX)

S1 Table List of *Hakea* species investigated and trait data. (DOCX)

S2 Table Eight fragment of *Hakea* DNA and their NCBI accession numbers used in *Hakea* phylogenetic reconstruction. (XLS)
