# Supplementary material for: Seed Size, Fecundity and Postfire Regeneration Strategy Are Interdependent in Hakea
Source: PLoS One. 2015 Jun 2;10(6):e0129027. doi: 10.1371/journal.pone.0129027 (PMC4455191; doi:10.1371/journal.pone.0129027)
Supplement: S1 Fig — (DOCX) [file pone.0129027.s002.docx]

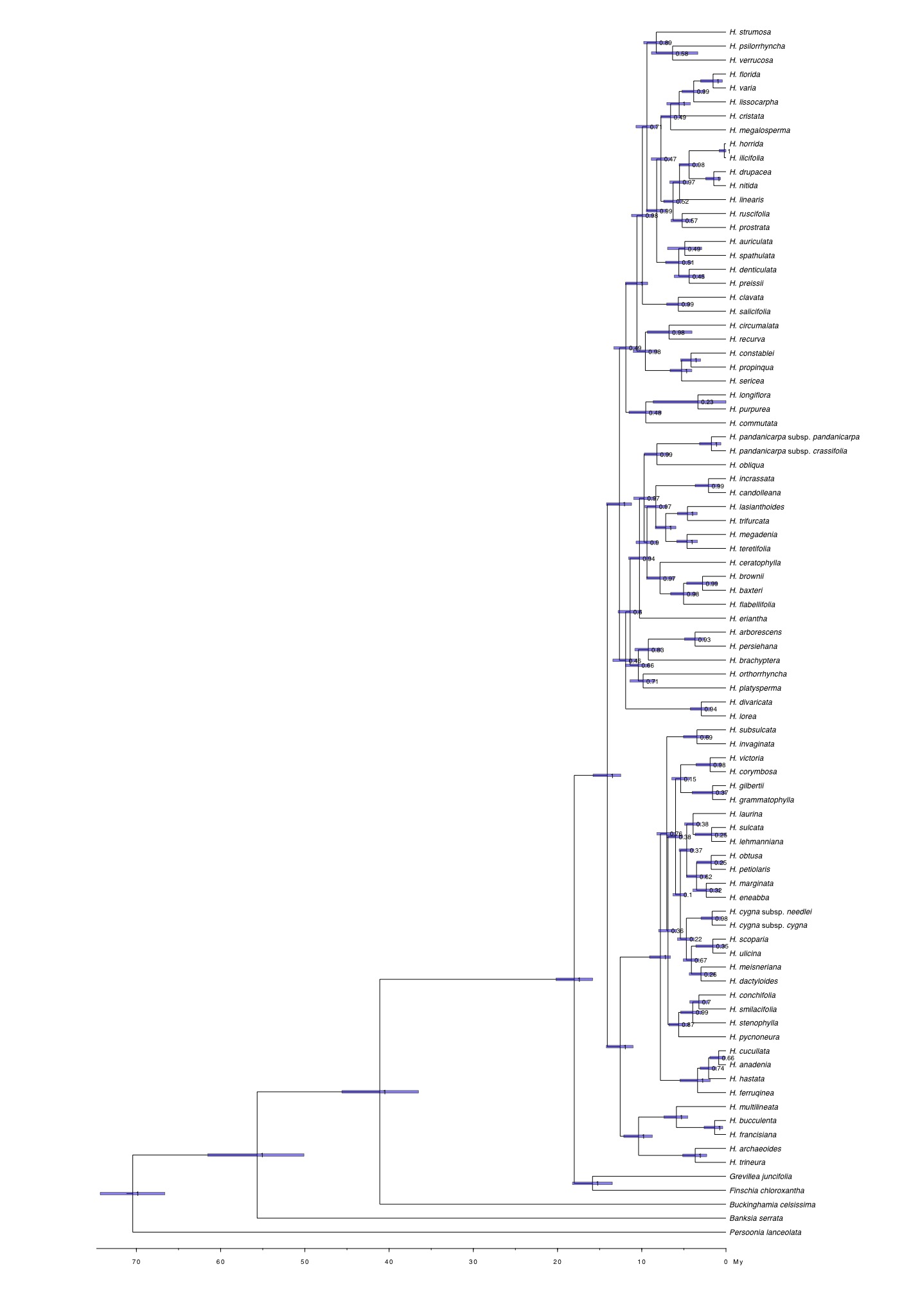


Figure S1. The *Hakea* maximum clade credibility dated phylogeny from the BEAST analysis with branch lengths relative to time. Posterior probability values for each clade are given above the branches. Horizontal purple bars represent 95% highest posterior density (HPD) intervals of divergence dates (mya) for each node.
