# Supplementary material for: Seed Size, Fecundity and Postfire Regeneration Strategy Are Interdependent in Hakea
Source: PLoS One. 2015 Jun 2;10(6):e0129027. doi: 10.1371/journal.pone.0129027 (PMC4455191; doi:10.1371/journal.pone.0129027)
Supplement: S1 Table — (DOCX) [file pone.0129027.s003.docx]

**Table. S1** List of *Hakea* species investigated and trait data. Fecundity: (average fruits stored per plant; 1 = 1-2, 2 = 3-5,3 = 6-10,4 = 11-50, 5 = 51-100, 6 = >100); Plant height: 1: <1 m, 2: 1-3 m, 3: > 3m. Serotiny: serotinous: seeds retained on plant > 3 years, Non/weakly serotinous: seeds retained on plant < 3 years ; “-”: data not available.

| Species | Seed size (mg) | Fruit size (g) | Fecundity | Serotiny | Plant height | Postfire regeneration |
| --- | --- | --- | --- | --- | --- | --- |
| *Hakea ambigua* | 11.2 | 1.96 | 4 | Serotinous | 2 | Nonsprouter |
| *Hakea anadenia* | 11.1 | 0.92 | 4 | Serotinous | 1 | Nonsprouter |
| *Hakea arborescens* | 61.0 | 5.36 | - | - | 3 | Nonsprouter |
| *Hakea archaeoides* | 27.0 | 1.93 | - | - | 3 | Resprouter |
| *Hakea auriculata* | 39.6 | 0.96 | 3 | Serotinous | 1 | Resprouter |
| *Hakea baxteri* | 39.9 | 9.14 | 3 | Serotinous | 2 | Nonsprouter |
| *Hakea brachyptera* | 35.2 | 7.24 | 4 | Serotinous | 1 | Nonsprouter |
| *Hakea brownii* | 81.9 | 17.00 | 1 | Serotinous | 2 | Resprouter |
| *Hakea bucculenta* | 12.4 | 1.86 | 6 | Serotinous | 2 | Nonsprouter |
| *Hakea candolleana* | 31.4 | 3.11 | 1 | Serotinous | 1 | Resprouter |
| *Hakea ceratophylla* | 23.1 | 1.35 | 2 | Serotinous | 1 | Resprouter |
| *Hakea circumalata* | 21.6 | 1.13 | 4 | Non/weakly Serotinous | 1 | Nonsprouter |
| *Hakea clavata* | 12.1 | 0.32 | 4 | Non/weakly Serotinous | 1 | Resprouter |
| *Hakea commutata* | 7.2 | 2.55 | 4 | Serotinous | 2 | Resprouter |
| *Hakea conchifolia* | 30.1 | 0.41 | 1 | Serotinous | 1 | Resprouter |
| *Hakea constablei* | 75.3 | 9.87 | - | - | 3 | Nonsprouter |
| *Hakea corymbosa* | 6.8 | 1.57 | 4 | Serotinous | 1 | Nonsprouter |
| *Hakea pandanicarpa subsp crassifolia* | 100.3 | 32.69 | 3 | - | 2 | Nonsprouter |
| *Hakea cristata* | 85.2 | 8.86 | 3 | Serotinous | 2 | Resprouter |
| *Hakea cucullata* | 29.0 | 4.43 | 4 | Serotinous | 2 | Nonsprouter |
| *Hakea cygna cygna* | 27.0 | 1.76 | 3 | Serotinous | 1 | Nonsprouter |
| *Hakea cygna needlei* | 35.5 | 5.27 | 2 | Serotinous | 1 | Nonsprouter |
| *Hakea dactyloides* | 23.1 | 3.97 | - | - | 3 | Nonsprouter |
| *Hakea denticulata* | 40.0 | 0.57 | 3 | Non/weakly Serotinous | 1 | Nonsprouter |
| *Hakea divaricata* | 39.0 | 0.64 | - | - | 3 | Nonsprouter |
| *Hakea drupacea* | 17.1 | 3.36 | 4 | Serotinous | 2 | Resprouter |
| *Hakea eneabba* | 19.1 | 0.92 | 3 | Serotinous | 1 | Resprouter |
| *Hakea eriantha* | 14.0 | 0.64 | - | Non/weakly Serotinous | 2 | Resprouter |
| *Hakea ferruqinea* | 6.7 | 1.93 | 5 | Serotinous | 2 | Nonsprouter |
| *Hakea flabellifolia* | 156.2 | 13.31 | 1 | Serotinous | 1 | Resprouter |
| *Hakea florida* | 30.4 | 6.06 | 4 | Serotinous | 2 | Resprouter |
| *Hakea francisiana* | 9.7 | 1.92 | 6 | Serotinous | 3 | Nonsprouter |
| *Hakea gilbertii* | 10.3 | 0.28 | 4 | Serotinous | 1 | Resprouter |
| *Hakea grammatophylla* | 11.6 | 0.7 | - | - | 2 | Nonsprouter |
| *Hakea hastata* | 7.4 | 0.69 | 4 | Serotinous | 2 | Nonsprouter |
| *Hakea horrida* | 11.1 | 0.86 | 4 | Serotinous | 1 | Resprouter |
| *Hakea ilicifolia* | 6.4 | 0.63 | 1 | Serotinous | 2 | Resprouter |
| *Hakea incrassata* | 60.1 | 10.32 | 2 | Serotinous | 1 | Resprouter |
| *Hakea invaginata* | 5.7 | 0.83 | 6 | Serotinous | 2 | Nonsprouter |
| *Hakea lasiocarpha* | 11.2 | 0.38 | 1 | Serotinous | 1 | Resprouter |
| *Hakea lasianthoides* | 20.1 | 0.41 | 4 | Non/weakly Serotinous | 2 | Nonsprouter |
| *Hakea laurina* | 20.2 | 0.32 | 5 | Serotinous | 3 | Nonsprouter |
| *Hakea lehmanniana* | 20.7 | 0.31 | 2 | Serotinous | 1 | Resprouter |
| *Hakea linearis* | 11.1 | 0.41 | 4 | Serotinous | 2 | Resprouter |
| *Hakea lissocarpha* | 23.9 | 0.59 | 2 | Serotinous | 2 | Resprouter |
| *Hakea longiflora* | 16.7 | 0.12 | 1 | Non/weakly Serotinous | 1 | Resprouter |
| *Hakea lorea* | 98.0 | 3.54 | - | - | 2 | Resprouter |
| *Hakea marginata* | 3.7 | 0.66 | 2 | Serotinous | 1 | Resprouter |
| *Hakea megadenia* | 5.2 | 0.69 | - | - | 2 | Resprouter |
| *Hakea megalosperma* | 109.1 | 13.43 | 1 | Serotinous | 1 | Resprouter |
| *Hakea meisneriana* | 5.3 | 0.42 | 4 | Serotinous | 2 | Nonsprouter |
| *Hakea multilineata* | 12.6 | 3.82 | 6 | Serotinous | 3 | Nonsprouter |
| *Hakea nitida* | 18.2 | 2.41 | 5 | Serotinous | 1 | Resprouter |
| *Hakea obliqua* | 26.9 | 7.11 | 4 | Serotinous | 2 | Nonsprouter |
| *Hakea obtusa* | 6.9 | 1.93 | 6 | Serotinous | 2 | Nonsprouter |
| *Hakea orthorrhyncha* | 43.8 | 5.01 | 6 | - | 2 | Resprouter |
| *Hakea pandanicarpa subsp pandanicarpa* | 100.0 | 32.69 | 3 | Serotinous | 2 | Nonsprouter |
| *Hakea pandanicarpa subsp crassifolia* | 100.3 | 32.69 | 3 | - | 2 | Nonsprouter |
| *Hakea persiehana* | 84.8 | 2.99 | - | - | 3 | Nonsprouter |
| *Hakea petiolaris* | 16.0 | 6.30 | 5 | Serotinous | 3 | Resprouter |
| *Hakea platysperma* | 508.8 | 40.45 | 3 | Serotinous | 2 | Nonsprouter |
| *Hakea preissii* | 12.5 | 0.42 | 5 | Non/weakly Serotinous | 2 | Nonsprouter |
| *Hakea propinqua* | 80.0 | 8.84 | - | - | 2 | Resprouter |
| *Hakea prostrata* | 60.0 | 1.41 | 2 | Non/weakly Serotinous | 2 | Resprouter |
| *Hakea psilorrhyncha* | 68.6 | 3.29 | 4 | Serotinous | 2 | Nonsprouter |
| *Hakea purpurea* | 18.0 | 3.24 | - | - | 1 | Resprouter |
| *Hakea pycnoneura* | 5.9 | 0.69 | 6 | Serotinous | 1 | Nonsprouter |
| *Hakea recurva* | 20.3 | 0.69 | 4 | Serotinous | 3 | Nonsprouter |
| *Hakea ruscifolia* | 30.0 | 0.87 | 2 | Non/weakly Serotinous | 2 | Resprouter |
| *Hakea salicifolia* | 20.2 | 3.67 | - | - | 3 | Nonsprouter |
| *Hakea scoparia* | 5.1 | 0.70 | 6 | Serotinous | 2 | Nonsprouter |
| *Hakea sericea* | 31.7 | 3.87 | 4 | - | 2 | Resprouter |
| *Hakea smilacifolia* | 9.9 | 0.06 | 4 | Serotinous | 1 | Nonsprouter |
| *Hakea spathulata* | 58.6 | 1.14 | 2 | Serotinous | 1 | Resprouter |
| *Hakea stenophylla* | 61.0 | 7.31 | 5 | Serotinous | 3 | Nonsprouter |
| *Hakea strumosa* | 62.7 | 14.45 | 3 | Serotinous | 1 | Resprouter |
| *Hakea subsulcata* | 5.4 | 0.66 | 4 | Serotinous | 2 | Nonsprouter |
| *Hakea sulcata* | 2.7 | 0.02 | 4 | Serotinous | 1 | Nonsprouter |
| *Hakea teretifolia* | 9.4 | 0.76 | - | Non/weakly Serotinous | 1 | Resprouter |
| *Hakea trifurcata* | 13.6 | 0.12 | 4 | - | 2 | Nonsprouter |
| *Hakea trineura* | 11.1 | 0.86 | - | - | 2 | Resprouter |
| *Hakea ulicina* | 6.4 | 0.83 | - | - | 2 | Nonsprouter |
| *Hakea varia* | 9.4 | 0.42 | 3 | Serotinous | 2 | Resprouter |
| *Hakea verrucosa* | 10.0 | 2.43 | 4 | Serotinous | 2 | Nonsprouter |
| *Hakea victoria* | 21.2 | 2.43 | 4 | Serotinous | 2 | Nonsprouter |
